# Candesartan and carvedilol for primary prevention of subclinical cardiotoxicity in breast cancer patients without a cardiovascular risk treated with doxorubicin

**DOI:** 10.1002/cam4.3956

**Published:** 2021-05-16

**Authors:** Myunhee Lee, Woo‐Baek Chung, Ji‐eun Lee, Chan‐Seok Park, Woo‐Chan Park, Byung‐Joo Song, Ho‐Joong Youn

**Affiliations:** ^1^ Division of Cardiology Department of Internal Medicine Daejeon St. Mary’s Hospital College of Medicine The Catholic University of Korea Seoul Korea; ^2^ Division of Cardiology Department of Internal Medicine Seoul St. Mary’s Hospital College of Medicine The Catholic University of Korea Seoul Korea; ^3^ Division of Oncology Department of Internal Medicine Seoul St. Mary’s Hospital College of Medicine The Catholic University of Korea Seoul Korea; ^4^ Division of Cardiology Department of Internal Medicine Bucheon St. Mary’s Hospital College of Medicine The Catholic University of Korea Seoul Korea; ^5^ Division of Breast Surgery Department of Surgery Seoul St. Mary's Hospital College of Medicine The Catholic University of Korea Seoul Korea; ^6^ Division of Breast‐Thyroid surgery Department of Surgery Bucheon St. Mary's Hospital College of Medicine The Catholic University of Korea Seoul Korea

**Keywords:** adrenergic beta‐antagonists, angiotensin receptor antagonists, anthracyclines, breast cancer, cardiomyopathies, prevention

## Abstract

**Background:**

There is no proven primary preventive strategy for doxorubicin‐induced subclinical cardiotoxicity (DISC), especially among patients without a cardiovascular (CV) risk. We investigated the primary preventive effect on DISC of the concomitant use of angiotensin receptor blockers (ARBs) or beta‐blockers (BBs), especially among breast cancer patients without a CV risk.

**Methods:**

A total of 385 patients who were scheduled for doxorubicin chemotherapy were screened. Among them, 195 patients of the study populations were included and were randomly divided into two groups [candesartan 4 mg q.d. vs. carvedilol 3.125 mg q.d.] and patients who were unwilling to take one of the medications were evaluated as controls. The primary outcomes were the incidence of early DISC (DISC developing within 6 months after chemotherapy), and late DISC (DISC developing only at least 12 months after chemotherapy).

**Result:**

Compared with the control group (8 out of 43 patients (18.6%)), only the candesartan group (4 out of 82 patients (4.9%)) showed a significantly lower incidence of early DISC (*p *= 0.022). Compared with the control group, the candesartan group demonstrated a significantly reduced decrease in left ventricular ejection fraction (LVEF) throughout the study period [−1.0% vs. −3.00 (*p <* 0.001) at the first follow‐up, −1.10% vs. −3.40(*p =* 0.009) at the second follow‐up].

**Conclusions:**

Among breast cancer patients without a CV risk treated with doxorubicin‐containing chemotherapy, subclinical cardiotoxicity is prevalent and concomitant administration of low‐dose candesartan might be effective to prevent an early decrease in LVEF. Further large‐scale, randomized controlled trials will be needed to confirm our findings.

## Graphical abstract

This study showed that low cumulative doses of doxorubicin were associated with the early development of subclinical cardiotoxicity in breast cancer patients without cardiovascular risk. Concomitant treatment with low‐dose candesartan may attenuate and even reverse early left ventricular systolic dysfunction in these patients.

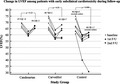

## 1 INTRODUCTION

Breast cancer is most commonly diagnosed among middle‐aged women and its incidence has increased consistently in Korea.^1^ Although the 5‐year survival rate among breast cancer patients in Korea increased over 90% in 2011–2015 due to advances in early detection and treatment,^1^ survivors of breast cancer are at a higher risk of cardiovascular disease‐related mortality than women without cancer.^2^, ^3^ Doxorubicin is the most widely used chemotherapeutic agent and have been known to be effective and increase survival in breast cancer patients.^4^ Their effectiveness, however, is hampered by the development of cardiotoxicity that leads to cardiomyopathy and end‐stage heart failure.^5^ The incidence of doxorubicin‐related cardiac dysfunction (defined as a reduction in ejection fraction of >10% below normal) has been reported as 16%, 32%, and 65% at total doxorubicin doses of 300, 400, and 550 mg/m^2^, respectively.^6^ Recent studies have suggested that doxorubicin‐induced cardiotoxicity may cause serious consequences even in patients with low cardiovascular risk (CV) and asymptomatic left ventricular (LV) dysfunction is associated with poor clinical outcome.^7^, ^8^

Studies have suggested that doxorubicin‐induced cardiotoxicity can be recovered when it is detected early, and appropriate treatment is started. ^9^, ^10^ As a result, recent studies have focused on early detection methods such as imaging (e.g., cardiac magnetic resonance (CMR), strain echocardiography and 3D echocardiography) and biomarkers (such as troponin and pro‐BNP). It is also well known that most doxorubicin‐induced cardiotoxicity occurs within the first year (mainly within 6‐months after chemotherapy).^9^ Traditionally, according to the time of onset, three types of cardiotoxicities have been recognized: (a) acute, occurring after a single dose, or a single course, of anthracyclines, with the onset of clinical manifestations within 2 weeks from the end of treatment; (b) early‐onset chronic, developing within 1 year. (c) late‐onset chronic, developing years, or even decades, after the end of chemotherapy. However, this classification was based on old (the early 1980s) small retrospective studies reporting occurrence of HF symptoms in childhood cancer survivor populations.^11^, ^12^ Until now, there is no definition of DISC (doxorubicin‐induced subclinical cardiotoxicity) according to time of onset.

Although clinical trials have proposed a potential protective effect with early medical interventions based on renin–angiotensin–aldosterone system inhibitors (RAASi) (including angiotensin‐converting enzyme(ACE) inhibitors and angiotensin receptor blockers (ARBs)) or beta‐blockers (BBs),^13^, ^14^, ^15^, ^16^, ^17^, ^18^, ^19^, ^20^, ^21^, ^22^ unfortunately, current recommendations(ESC,^23^ ASCO,^24^ ESMO^25^ ) are not sufficient to support clear evidence‐based recommendations on preventive management strategies for breast cancer patients with chemotherapy‐induced cardiotoxicity owing to inconsistent results and, diversities in patient populations with different types of cancer, study designs and treatment regimens. A recent study showed that even a low cumulative dose of doxorubicin chemotherapy was associated with early subclinical CV events.^26^ Moreover, based on data from the Korea breast cancer registry, the proportion of early breast cancer continues to increase, and the proportion of young breast cancer patients without a CV risk is higher in Korea than in the West.^27^ Still, many physicians are reluctant to prescribe cardioprotective medications due to fear of drug side effects such as hypotension and electrolyte imbalance and lack of evidence in breast cancer patients without CV disease. But some physicians prescribed low‐dose candesartan or carvedilol for off‐label use to prevent DISC even though patients did not have CV diseases such as hypertension. Furthermore, little is known about the incidence of DISC and preventive strategies for DISC among breast cancer patients without a CV risk.

We, therefore, conducted this study to determine whether early detection of DISC and preventive administration of low‐dose ARBs or low‐dose beta‐blockers with doxorubicin chemotherapy will mitigate the decrease in LV systolic function in breast cancer patients without a CV risk.

## 2 METHODS

### 2.1 Study design and population

**FIGURE 1:**
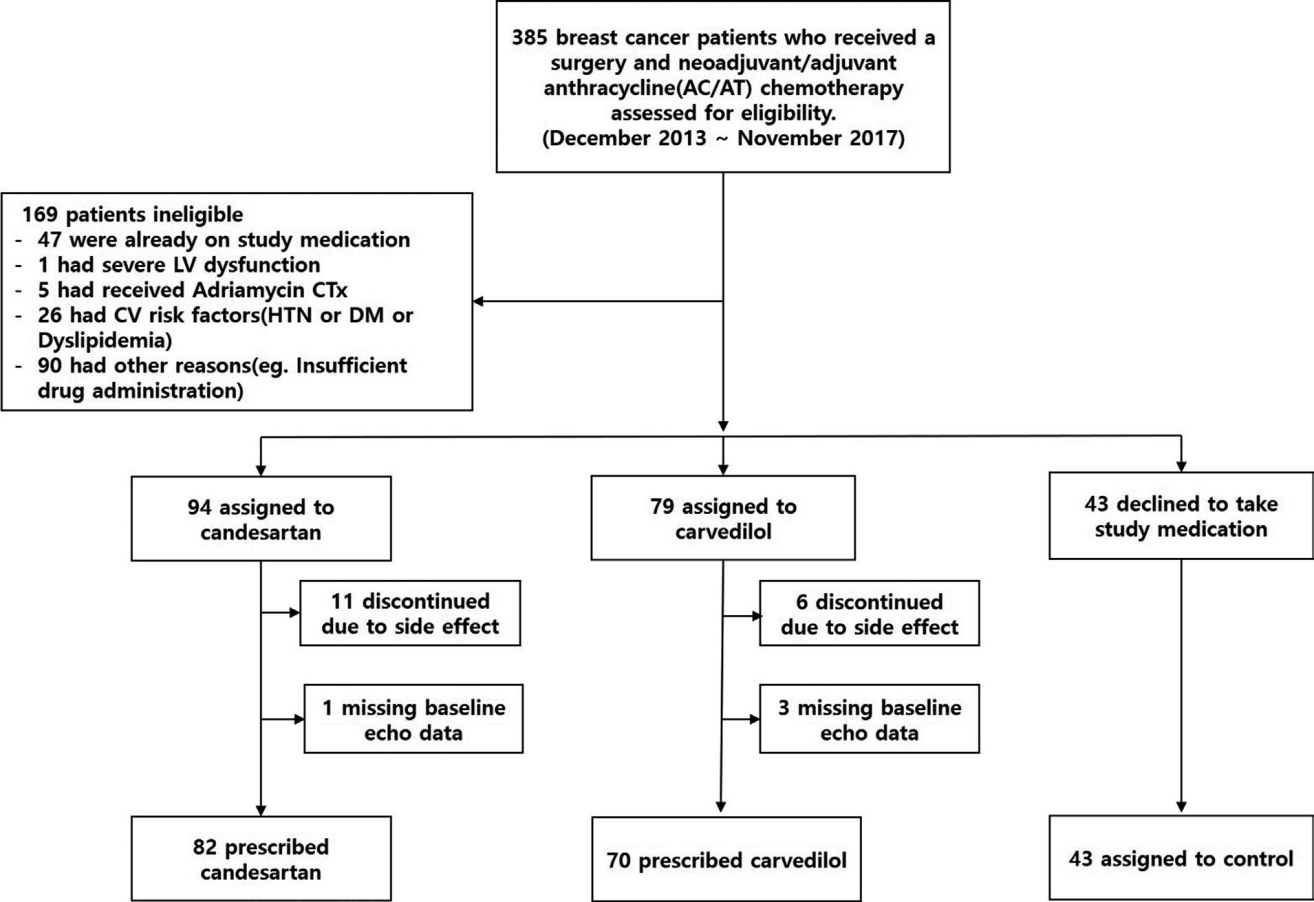
Screening process of the study. From December 2013 to November 2017, a total of 243 eligible patients were enrolled in the study and were randomized to the candesartan or carvedilol group. Patients who declined to take either medication were assigned to the control group. AC, adriamycin (doxorubicin) and cyclophosphamide; AT, adriamycin (doxorubicin) and taxol (paclitaxel); CV, cardiovascular; DM, diabetes mellitus; HTN, hypertension; LV, left ventricle

SAVE HEART (proSpective registry for prediction And preVEntion of cHEmotherapy‐induced cARdiotoxicity in patients with breasT cancer) was a randomized phase III study to compare the role of candesartan and carvedilol in preventing cardiotoxicity (including both subclinical cardiotoxicity and overt heart failure) among breast cancer patients. This study is a post‐hoc analysis of SAVE HEART study to evaluate the effect of candesartan or carvedilol compared with the control in preventing DISC in patients without a CV risk. From December 2013 to November 2017, 385 patients with breast cancer who underwent surgery and were scheduled for neoadjuvant or adjuvant chemotherapy with doxorubicin were screened in the study. A total of 195 patients were randomly allocated on a 1:1 basis to receive treatments with either candesartan 4 mg q.d. or carvedilol 3.125 mg q.d. (Figure 1). The randomization was performed by a computer‐generated random table. Forty‐three patients who consented to participation in this study but declined to take either medication were assigned to the control group. The study complied with the Declaration of Helsinki and was approved by the Institutional Review Board (IRB) of The Catholic University of Korea (IRB number: XC10RIMI0091S). All the patients were informed about the objectives, protocol, alternative treatment options and possible side effects. All the patients provided written informed consent to participate in the study. The standard chemotherapy protocol comprised either 4 cycles of cyclophosphamide 600 mg/m^2^ and doxorubicin 60 mg/m^2^ every 21 days (with a total cumulative dose of 240 mg/m^2^) or 6 cycles of docetaxel 75 mg/m^2^ and doxorubicin 50 mg/m^2^ every 21 days (with a total cumulative dose of 300 mg/m^2^). The choice of the protocol was left to the oncologist's discretion.

Inclusion criteria were female sex, age over 18 years, a diagnosis of breast cancer with indications for adjuvant or neoadjuvant doxorubicin chemotherapy (either AC (adriamycin‐cyclophosphamide) or AT (adriamycin‐docetaxel) chemotherapy only), and normal LV systolic function (left ventricular ejection fraction (LVEF) ≥50%). Exclusion criteria were other chemotherapy regimens beside AC or AT chemotherapy; LV systolic dysfunction (LVEF<50%); heart failure symptoms; significant coronary artery disease, valvular heart disease, or arrhythmia; prior (within 4 weeks of study initiation) use of study medications; prior history of doxorubicin or trastuzumab chemotherapy; and a diagnosis of hypertension, diabetes or dyslipidemia, which are known risk factors for doxorubicin‐induced cardiotoxicity. Details on patient inclusion/exclusion criteria are listed in the supplemental material. (Table S1).

### 2.2 Study procedure

All eligible patients underwent a baseline transthoracic echocardiogram (TTE) and routine laboratory tests before receiving chemotherapy. Baseline TTE was performed within 3 months before initiation of chemotherapy, and follow‐up TTE was performed within 3 months after completion of chemotherapy. Patients received the study medication within 1 month after the initiation of chemotherapy and were maintained on the medication throughout chemotherapy without a titrating dose. If cardiotoxicity was confirmed or suspected heart failure occurred, the study medication continued according to the physician's decision. The first TTE follow‐up was performed after completion of doxorubicin chemotherapy and before 6 months of doxorubicin chemotherapy (median (25th‐75th percentile) 2.0 (2.0–5.0) months). The second TTE follow‐up was performed at least 12 months after the completion of doxorubicin chemotherapy (median (25th–75th percentile) 16.0 (11.0–18.0) months). Two experienced sonographers performed the TTE. All TTE images were reviewed and interpreted by two experienced, board‐certified echocardiography specialists with the use of available machines from our hospital (GE vivid 7, GE vivid E‐9 (GE Healthcare, Fairfield, USA), Philips IE 33 (Philips Andover, Andover, USA)). Both sonographers and echocardiography specialists are blind to the study population. LVEF was measured by the modified Simpson's method via apical 4‐ and 2‐chamber views. LV diastolic function was assessed by the E/A ratio, E/e’ ratio, TR velocity, mitral or lateral annulus e velocity determined by pulsed‐wave doppler and left atrium size. DISC was defined when the LVEF decreased more than 10% from the baseline or below 50% at the follow‐up without symptoms or signs of congestive heart failure.^25^

### 2.3 Study endpoints

The primary outcome was the incidence of early DISC (DISC developing within 6 months after chemotherapy) and late DISC (DISC developing only at least 12 months after chemotherapy). Secondary outcomes included changes in LVEF from baseline and after doxorubicin chemotherapy and the effects of candesartan or carvedilol on diastolic dysfunction (defined if more than half of the following criteria were satisfied: average E/e’ >14, septal e’ velocity <7 cm/s or lateral e’ velocity <10 cm/s, TR velocity >2.8 m/s and left atrium (LA) volume index >34 ml/m^2^), changes in LA size and change in LV size.

### 2.4 Statistical analysis

Continuous variables are expressed as the mean ±SD when normally distributed or as the median (interquartile range) if the hypothesis of distribution normality was rejected. Categorical data are presented as absolute value and percentage and were compared by using the chi‐square test or Fisher's exact test. Continuous variables were compared by paired *t*‐test (Mann–Whitney U test) or ANOVA (Kruskal–Wallis test) as appropriate. The primary outcomes were analyzed by means of Fisher's exact test for pairwise comparisons between treatments; a *p* value of 0.025 was considered to indicate statistical significance (accounting for Bonferroni correction). Otherwise, a *p* value of <0.05 was considered statistically significant, and the confidence interval (CI) was 95%. The statistical analysis was performed using MedCalc 19.1.3 (MedCalc Software Ltd., Acacialaan 22 8400 Ostend, Belgium).

## 3 RESULTS

The baseline characteristics of the patient population are summarized in Table 1. All patients were female, young (mean 47.5 ±8.7 years old), non‐smokers, had a normal body mass index (22.4 kg/m^2^, median), were normotensive, had no prior history of coronary artery disease, and received a low cumulative dose of doxorubicin (240 mg/m^2^(median)). The baseline characteristics of the study population were statistically balanced among the study groups (Table 1). LVEF at baseline was similar among the study groups.

**TABLE 1:** Baseline characteristics of study population

Total N = 195	CDRT N = 82	CVDL N = 70	Control N = 43	*p* value
Age at recruitment(years)	47.8 ± 8.7	46.6 ± 7.6	48.5 ± 10.4	0.507*
BMI (kg/m^2^)	22.7 ± 3.1	22.7 ± 2.6	23.1 ± 2.8	0.667*
SBP (mmHg)	115.2 ± 10.3	114.9 ± 10.4	113.7 ± 16.2	0.883*
DBP (mmHg)	72.6 ± 9.3	71.6 ± 8.6	71.4 ± 9.7	0.749*
HR (b.p.m)	84.2 ± 13.4	83.2 ± 12.2	80.7 ± 11.1	0.343*
Serum Cr (mg/dL)	0.71 ± 0.10	0.68 ± 0.08	0.67 ± 0.08	0.066*
Serum Hb (g/dL)	13.1(12.2–14.0)	13.1(12.6–13.7)	12.8(12.1–13.6)	0.353*
Fasting glucose	95.5(89.0–102.0)	94.5(91.0–103.0)	97.0(90.3–103.3)	0.898*
Trastuzumab (%)	15(18.3%)	10(14.3%)	3(7.0%)	0.230*
Radiation (%)	68(82.9%)	50(71.4%)	35(81.4%)	0.198*
Cumulative Adriamycin dose(mg/BSA)	240 (240.0–240.0)	240 (240.0–240.0)	240 (234.0–240.0)	0.564*
Reason of CTx				
Neoadjuvant	21(25.6)	10(14.3)	7(16.3)	
Adjuvant	61(74.4)	59(84.3)	36(83.7)	
Palliative	0(0)	1(1.3)	0(0)	
CTx regimen				0.741**
AC	70(85.4)	62(90.8)	36(83.7)	
AT	12(14.6)	8(9.2)	7(6.3)	
Stages				
I	39(47.6)	34(47.4)	17(39.5)	
II	31(37.8)	27(40.8)	18(41.9)	
III	12(14.6)	8(10.5)	8(18.6)	
IV	0(0%)	1(1.3)	0(0)	

Data are presented as mean ±SD or median (interquartile range) or n (%).

Abbreviations: AC, adriamycin‐cyclophosphamide; AT, adriamycin‐docetaxel; BMI, body mass index; BSA, body surface area; CDRT, candesartan; Cr, creatine; CTx, chemotherapy; CVDL, carvedilol; DBP, diastolic blood pressure; DM, diabetes mellitus; Hb, haemoglobin; HR, heart rate; HTN, hypertension; SBP, systolic blood pressure.

* *p* value calculated by ANOVA (or Kruskal–Wallis test).

** *p* value calculated by Fisher's exact test.

### 3.1 Primary outcomes

Early DISC developed in 4 patients (4.9%) in the candesartan group, 6 patients (8.6%) in the carvedilol group and 8 patients (18.6%) in the control group (*p =* 0.041; incidence of early subclinical cardiotoxicity at first F/U in Table 2). Compared with the control group, the candesartan group showed a statistically significant lower incidence of early DISC (*p =* 0.022, Table 2), whereas the carvedilol group showed a nonsignificant reduction in the incidence of early DISC (*p =* 0.145, Table 2). Notably, at the time of the second follow‐up, all 4 patients who had developed early DISC in the candesartan group had recovered, but 3 out of 6 (50%) patients in the carvedilol group and 3 out of 8(37.5%) patients in the control group remained in a state of subclinical cardiotoxicity (incidence of early subclinical cardiotoxicity at second F/U in Table 2). Additionally, there was a significant difference in the incidence of late DISC among the study groups and the control group (3 (3.8%), 2 (3.1%), and 5 (14.3%), respectively, *p =* 0.046; Table 2). Among patients who developed early DISC, three patients in the candesartan group, four patients in the carvedilol groups and 6 patients in the control group underwent radiation therapy. Among patients who developed late DISC, 2 patients in the candesartan group, 2 patients in the carvedilol groups, and 4 patients in the control group underwent radiation therapy.

**TABLE 2:** Primary and secondary outcomes

Total N = 195	CDRT N = 82	CVDL N = 70	Control N = 43	Overall *p* value	CDRT vs. Control *p* Value	CVDL vs. Control *p* valve
Incidence of Early subclinical cardiotoxicity						
First F/U	4(4.9)	6(8.6)	8(18.6)	0.041**	0.022**	0.145**
Second F/U	0(0)	3(4.3)	3(7.0)			
Incidence of Late subclinical cardiotoxicity						
	3(3.8)	2(3.1)	5(14.3)	0.046**	0.105**	0.093**
**Sequential LVEF, %**				0.003****		
Baseline	64.5 (63.7,65.2)	65.0 (63.0 – 66.1)	64.0 (62.8 – 66.7)	0.66*		
First F/U	63.4 (61.1–65.9)	62.9 (61.0 – 64.0)	60.2 (58.4–63.0)	<0.001*		
Difference of LVEF at first f/u	−1.00 (−3.90–−1.80)	−1.65 (−3.60 – −0.00)	−3.00 (−5.63–−1.73)	<0.001*	<0.001***	0.011***
Second F/U	63.3 (62.7,63.9)	62.8 (62.0,63.7)	61.3 (59.2–62.9)			
Difference of LVEF at second f/u	−1.10 (−3.73–1.63)	−2.30 (−4.18 – 0.85)	−3.40 (−6.45–−1.15)	0.009*	0.002***	0.055***

Data are presented as mean (95% CI) or median (interquartile range) or n (%).

Abbreviations: CDRT, candesartan; CI, confidence interval; CTx, chemotherapy; CVDL, carvedilol; LVEF, left ventricular ejection fraction; OR, odds ratio.

* *p* value calculated by ANOVA (or Kruskal–Wallis test).

** *p* value calculated by chi‐square test (Fisher's exact test).

*** *p* value calculated by t test. (Mann–Whitney U test).

**** *p* value calculated by repeated measure analysis of variance.

### 3.2 Secondary outcomes

**FIGURE 2:**
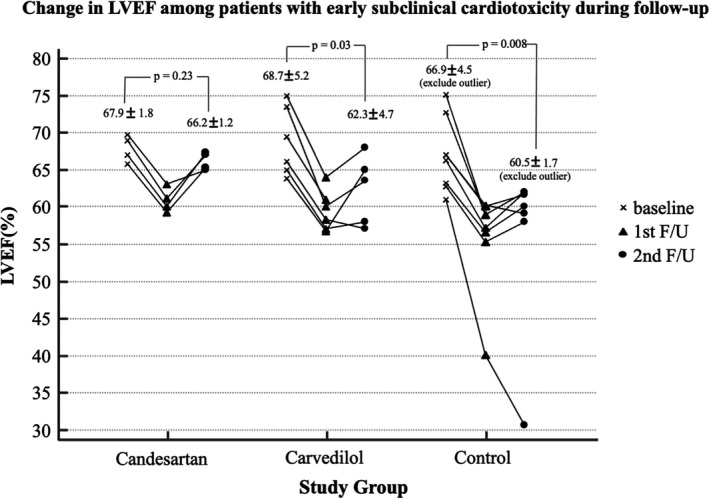
Change in LVEF in patients with subclinical cardiotoxicity during follow‐up. Among patients who developed cardiotoxicity, both those in the carvedilol and the control groups showed a significant decrease in LVEF from baseline to the end of the study. However, those in the candesartan group alone showed an insignificant change in LVEF from baseline to the end of the study. LVEF, Left ventricular ejection fraction; F/U, follow‐up

Baseline LVEF was similar among the study groups (*p* = 0.55). At the first follow‐up, the decrease in LVEF was significantly different among the study groups (−1.00%, −1.65% and −3.00%, respectively, overall *p <* 0.001; Table 2). Both study groups showed a significantly smaller change in LVEF than the control group at the first follow‐up period (*p <* 0.001 and *p <* 0.001, respectively; Table 2). At the second follow‐up, the decrease in LVEF was also significantly different among the study groups. (−1.10%, −2.30% and −3.40%, respectively, overall *p =* 0.009; Table 2). However, only the candesartan group showed a statistically lower decrease in LVEF (*p =* 0.002). Among the patients who developed subclinical cardiotoxicity, all except those in the candesartan group showed statistically significant changes in LVEF during the follow‐up period (Figure 2). Furthermore, the effect of candesartan on LVEF was consistent across the predefined subgroups when patients were stratified according to age, body mass index, cumulative dose, or radiation. (Figure S1), but the effect of carvedilol on LVEF was inconsistent across the predefined subgroups (Figure S2). Because only a small percentage of the population (14.4%) received trastuzumab followed by chemotherapy, we did not conclude the effect of candesartan or carvedilol on LVEF according to trastuzumab therapy. Baseline parameters of diastolic function were suggestive of normal with an E/A ratio close to 1, no LA enlargement, an E/e’ less than 14 and no significant TR (tricuspid regurgitation). No significant changes in the diastolic parameters were observed in any of the groups after chemotherapy. There was no difference in the parameters of diastolic dysfunction and LV size after chemotherapy among the study groups (Table S2.).

### 3.3 Safety outcomes

Generally, the study medications were well tolerated and no differences in the incidence of side effects occurred across groups during follow‐up (*p =* 0.45; Table S3). No serious side effects were observed in any study group. The most common side effect was gastrointestinal (GI) trouble (Table S3).

## 4 DISCUSSION

In contemporary cardio‐oncology practice, physicians often neglect risk assessment and monitoring of cardiotoxicity for cancer patients with a low CV risk profile despite guidelines recommended that physicians assess the potential cardiotoxicity risk of all cancer patients scheduled to receive cardiotoxic chemotherapy and implant individual surveillance programs for timely diagnosis and pre‐treatment of cardiotoxicity before chemotherapy. In this study, we investigated the effectiveness of low‐dose candesartan or carvedilol in preventing DISC in female breast cancer patients without a CV risk. The main findings of this study can be summarized as follows: (a) Subclinical cardiotoxicity after doxorubicin chemotherapy is observed frequently, even in patients without a CV risk. (b) pretreatment with low‐dose candesartan may prevent the early decrease in doxorubicin‐induced LV systolic dysfunction and that the protective effect of candesartan remained significant even 1 year after the completion of doxorubicin‐containing chemotherapy. To the best of our knowledge, this is the first clinical trial to include only breast cancer patients without a CV risk treated with doxorubicin chemotherapy, which confirms the protective effect of low‐dose candesartan on DISC.

The activation of the renin‐angiotensin‐aldosterone system promotes LV remodeling regardless of its etiology and eventually leads to systolic or diastolic heart failure. Thus, RAASi have become the cornerstone therapy for heart failure, but there are conflicting results on the potential cardioprotective role of RAASi in the prevention of doxorubicin‐induced cardiotoxicity. In an animal study, chronic administration of doxorubicin resulted in cardiotoxicity and was associated with a 2‐fold increase in plasma renin activity, the enzyme responsible for initiating the renin‐angiotensin system.^28^, ^29^ Evidence suggests that gender differences exist in terms of renin–angiotensin–aldosterone system function. Miller et al. showed that women achieved considerably lower angiotensin II sensitivity than men at lower dosages, suggesting that women may require lower dosages of ARB than men.^30^ Hudson et al. investigated the efficacy of ACE inhibitors and ARBs between men and women among patients with congestive heart failure. In a cohort study, they showed that women prescribed ARBs had better survival than those prescribed ACE inhibitors.^31^ ARBs have demonstrated superior safety profiles to those of ACE inhibitors and beta‐blockers, and the risk of discontinuation due to adverse events is reduced by 29% with ARB treatment.^32^

This study demonstrated several important findings. First, we found that the incidence of early subclinical cardiotoxicity was relatively high (18.6%), although the study population consisted of breast cancer patients without a CV risk who received a low cumulative dose of doxorubicin. In a previous study, our group reported that subclinical cardiotoxicity was common in young adult breast cancer at a relatively low cumulative dose of doxorubicin.^33^ In this study, we also showed that contemporary doxorubicin chemotherapy is associated with a modest absolute reduction in LVEF (−3.0%). These findings were similar to prior studies (−2.6% in the PRADA^17^ trial, −3.28% in the OVERCOME^20^ trial) even though our study included only patients without a CV risk (although we excluded smokers and those who had been diagnosed with hypertension or diabetes, the PRADA^17^ study included 18.8% smokers, 8.3% hypertension patients and 2% diabetic patients, while the OVERCOME^20^ study included 18.9% smokers, 15.6% hypertension patients and 4.4% diabetic patients). Furthermore, in our study LV systolic function deteriorated early and persisted abnormally until 1 year after the initiation of the low cumulative dose of doxorubicin chemotherapy. Our findings suggest that special concerns, including precise monitoring and appropriate interventions, should be taken into consideration to prevent DISC among breast cancer patients even if they have no CV risk factors. However, long‐term follow‐up is needed to demonstrate whether DISC is associated with overt heart failure or poor clinical outcomes.

Second, as shown in previous trials,^17^, ^20^ concomitant administration of candesartan was significantly effective in preventing the decrease in LVEF after chemotherapy. Moreover, we found that the protective effect of candesartan was maintained over 1 year after the end of doxorubicin chemotherapy, while the protective effect of carvedilol was reduced after 1 year of the completion of chemotherapy. The protective effect of candesartan against a decrease in LVEF was achieved despite prescribing the smallest dose of candesartan (4 mg daily). Additionally, the candesartan‐treated group presented with a lower incidence of early subclinical cardiotoxicity and a greater preventive effect against the reduction in LVEF than the carvedilol group. Particularly, among patients who developed early subclinical cardiotoxicity, all of the patients in the candesartan group recovered from cardiotoxicity at the end of the study and showed a nonsignificant difference in LVEF between baseline and the end of the study (Figure 2). These results suggest that candesartan has not only a preventive effect on the development of DISC but also a therapeutic effect on DISC. In this study, we prescribed candesartan at the early phase after chemotherapy (within 1 month after chemotherapy) and identified the asymptomatic decline of LVEF at the early stage (median 2 months after completion of doxorubicin chemotherapy) and maintained candesartan until LVEF recovered. As shown in Figure 2, all patients who developed cardiotoxicity had a preserved LVEF (LVEF >55%). Early detection at the asymptomatic phase and early initiation of treatment might have played a crucial role in preventing and recovering from cardiotoxicity. A possible explanation for the discrepancy between the effect of candesartan and carvedilol is that the prescribed dose of carvedilol was too small to show any clinical effects, and unlike candesartan, the maximally tolerated dose of carvedilol based on heart rate may be beneficial for averting LV systolic dysfunction related to doxorubicin. Therefore, we cannot rule out possible protective effects of higher doses or other classes of beta‐blockers.

Third, it has been reported that left ventricular diastolic dysfunction occurs earlier than systolic dysfunction after doxorubicin chemotherapy.^34^ Avila et al. showed that carvedilol was associated with a decrease in LV end‐diastolic diameter and prevention in developing diastolic dysfunction.^15^ In this study, we did not notice any difference in LV size or parameters related to LV diastolic dysfunction before and after chemotherapy among the study groups (Table S2). There are several potential reasons for the negative effect of diastolic dysfunction in our study. First, in the CECCY trial, patients were more susceptible to diastolic dysfunction than in our study because those patients had a higher prevalence of cardiovascular comorbidities and were older and more obese. Second, in our study, we prescribed a much lower dose of carvedilol (3.125 mg) than the CECCY trial (18.5 mg). The dose of carvedilol may be insufficient to produce beneficial effects on LV diastolic function. Third, the traditional diastolic parameters used in our study might not be appropriate to assess chemotherapy‐related cardiac dysfunction. Some studies were unable to identify traditional diastolic parameters as early predictors for doxorubicin cardiotoxicity.^34^, ^35^ Given the limited data available, the 2014 ASE (American Society of Echocardiography) guidelines emphasize that the use of conventional diastolic parameters is of uncertain value in the assessment of chemotherapy‐induced cardiotoxicity.

### 4.1 Study limitations

There are several limitations in this study. First, this study was an open‐label, partially randomized trial (candesartan vs. carvedilol) but the control group was not randomly assigned. This unconventional study design introduces the possibility of selection bias. Also, this study has a relatively small sample size (approximately 200 patients). Nevertheless, this study included only female breast cancer patients without a CV risk. Due to its unique study design and patient population, we expect that the results of our study could form a scientific basis for the management of breast cancer patients who require doxorubicin chemotherapy. The results of this study should be confirmed by large‐scale, randomized controlled trials. Second, we used 2D echocardiography by measuring LVEF. Other echocardiographic modalities such as 2D/3D speckle echocardiography, 3D echocardiography and CMR, might be more sensitive in detecting subclinical myocardial dysfunction and should be considered for future studies. Third, we did not include biomarkers such as troponin I/T or pro‐BNP to detect early subclinical cardiotoxicity because they were not yet part of standard care at the time of the study; as a result, only one out of every five patients underwent biomarker study. Fourth, this study demonstrates that neurohormonal therapy can prevent small drops in LVEF in breast cancer patients exposed to anthracycline‐based chemotherapy. However, it is not clear if this has any impact on long‐term clinical outcomes. Fifth, we did not analyze the effect of hormone or radiation therapies on DISC. They have known risk factors for chemotherapy‐related overt heart failure, but the relationship between hormone or radiation therapies and DISC is still unknown, and more studies will be needed. Sixth, this study was conducted only in Korean breast cancer women, making our results difficult to generalize to other types of cancer patients or other ethnic groups.

## 5 CONCLUSIONS

In breast cancer patients without a CV risk who treated with doxorubicin‐containing chemotherapy, subclinical cardiotoxicity was prevalent and concomitant administration of low‐dose candesartan demonstrated a lower incidence of doxorubicin‐induced subclinical cardiotoxicity (DISC) compared with control groups. The findings of this study suggest that concomitant administration of low‐dose candesartan with doxorubicin‐containing chemotherapy might be effective in preventing an early decrease in LVEF among breast cancer patients without a CV risk. The results of the present study could provide physicians clinical insights for identifying and managing DISC among breast cancer patients without a CV risk. However, further large‐scale, randomized controlled trials will be needed to confirm our findings and to check the long‐term consequences of preventing DISC.

## CONFLICTS OF INTEREST

There is no conflict of interest to disclose from any authors.

## AUTHORS’ CONTRIBUTIONS

Myunhee Lee: Conceptualization, Investigation, Formal analysis, Writing‐ Original Draft, Review & Editing, Visualization. Woo‐Baek Chung: Conceptualization, Methodology, Investigation, Data curation, Writing‐ Review & Editing, Supervision. Ji‐eun Lee: Investigation, Writing‐ Review & Editing. Chan‐Seok Park: Investigation, Writing‐ Review & Editing. Woo‐Chan Park: Investigation, Data curation, Writing‐ Review & Editing. Byung‐Joo Song: Investigation, Data curation, Writing‐ Review & Editing. Ho‐Joong Youn: Conceptualization, Methodology, Data curation, Writing‐ Review & Editing, Project Administration.

## ETHICAL APPROVAL

This study was approved by the Institutional Review Board of The Catholic University of Korea, and informed consent was obtained according to the Declaration of Helsinki.

## Supporting information

Supplementary Material

## Data Availability

The data that support the findings of this study are available from the corresponding author upon reasonable request.
