# Supplementary material for: Candesartan and carvedilol for primary prevention of subclinical cardiotoxicity in breast cancer patients without a cardiovascular risk treated with doxorubicin
Source: Cancer Med. 2021 May 16;10(12):3964–73. doi: 10.1002/cam4.3956 (PMC8209607; doi:10.1002/cam4.3956)
Supplement: Supplementary file 1 — Supplementary Material [file CAM4-10-3964-s001.docx]

**Supplemental Material**

**TABLE S1 Details on Patients Inclusion and Exclusion Criteria**

**Inclusion criteria**

Scheduled for adjuvant or neoadjuvant doxorubicin chemotherapy (either AC (adriamycin-cyclophosphamide) or AT (adriamycin-docetaxel))

Age over 18 years

Eastern Cooperative Oncology Group (ECOG) performance status 0–1

Serum creatinine <1.6 mg/dL or estimated glomerular filtration rate (eGFR) ≥ 60 ml/min/1.73 m2

Systolic blood pressure $\geq$ 90 mmHg and <160 mmHg

Left ventricular ejection fraction ≥ 50%

Study medication initiated within 1 month before and after initiation of chemotherapy

**Exclusion criteria**

Other chemotherapy regimens besides AC or AT chemotherapy

Hypotension, defined as systolic blood pressure < 90 mmHg

Prior use of doxorubicin or trastuzumab-containing chemotherapy regimens

Prior malignancy requiring chemotherapy or radiotherapy

Symptomatic heart failure

Systolic LV dysfunction (left ventricular ejection fraction < 55%)

Clinically significant coronary artery disease, valvular heart disease, significant arrhythmias, or conduction delays

Bradycardia, defined as heart rate < 50 beats per minute

Diabetes(HbA1c ≥ 6.5%(48 mmol/mol), fasting glucose ≥ 7.0 mmol/l (126 mg/dl) or on antidiabetic medication(per os or insulin)

Hypertension(SBP ≥ 140 and/or DBP ≥ 90 or on antihypertensive medication)

Dyslipidemia(total cholesterol ≥240 mg/dL, LDL ≥ 160 mg/dL, HDL ≤ 40 mg/dL, triglyceride ≥ 200 or on lipid-lowering medication)

Treatment with angiotensin-converting enzyme inhibitor, angiotensin receptor blocker, beta-blocker or mineralocorticoid receptor antagonists within the last 4 weeks prior to study start

**TABLE S2** Secondary outcomes. (diastolic function and LV size)

| **Total**  **N=195** | **CDRT**  **N = 82** | **CVDL**  **N = 70** | **Control**  **N=43** | | **P value** | |
| --- | --- | --- | --- | --- | --- | --- |
| **Diastolic function** |  |  |  | |  | |
| Baseline (No. (%)) | 0 | 0 | 1 | |  | |
| After CTx (No. (%)) | 0 | 0 | 1 | |  | |
| E/A |  |  |  | |  | |
| Baseline | 1.21  (0.87-1.55) | 1.26  (0.88-1.48) | 1.26  (0.88-1.45) | | ^*^0.78 | |
| 1^st^ F/U | 1.17  (0.86-1.35) | 1.23  (0.85-1.42) | 1.19  (0.86-1.40) | | ^*^0.85 | |
| E/E’ |  |  |  | |  | |
| Baseline | 7.33±1.58 | 6.90±1.89 | 6.90(5.98-8.30) | | ^*^0.22 | |
| 1^st^ F/U | 7.60±1.79 | 7.51± 1.57 | 7.50(6.28-98.98) | | ^*^0.74 | |
| LA size (LAVI) |  |  |  | |  | |
| Baseline | 20.3(17.0-22.4) | 18.6±8.84 | 22.2±6.27 | | ^*^0.14 | |
| 1^st^ F/U | 21.3(18.4-25.5) | 20.9(17.5-25.0) | 19.9(18.5-24.9) | | ^*^0.85 | |
| **LV size (mm)** |  |  |  | |  | |
| LVEDD |  |  |  | |  | |
| Baseline | 46.4±3.79 | 45.3±3.53 | 46.3±4.78 | | ^*^0.16 | |
| 1^st^ F/U | 46.7±3.52 | 46.1±3.44 | 46.8±3.19 | | ^*^0.48 | |
| LVESD |  |  |  | |  | |
| Baseline | 28.2±3.31 | 27.8±2.97 | 29.0(26.4- 30.8) | | ^*^0.47 | |
| 1^st^ F/U | 29.2±3.19 | 28.7 ±3.00 | 29.7±3.54 | | ^*^0.25 | |
|  |  |  |  |  | |  |

CDRT, candesartan; CVDL, carvedilol; CTx, chemotherapy; LA, left atrium; LAVI, left atrial volume index; LVEDD, left ventricle end diastolic dimension; LVESD, left ventricle end systolic dimension.

Data are presented as mean ±SD or median (interquartile range). ^*^P value calculated by ANOVA (or Kruskal-Wallis test).

**TABLE S3** Side effects of study medications.

| **Event, no. of patients (%)** | **CDRT**  **(N=94)** | **CVDL**  **(N=79)** | **P value** |
| --- | --- | --- | --- |
| Total (%) | 11(11.7) | 6(7.6) | ^*^0.45 |
| Symptomatic hypotension | 1 | 2 |  |
| Dizziness | 1 | 0 |  |
| Shortness of breath | 0 | 0 |  |
| GI trouble | 8 | 4 |  |
| Palpitations | 1 | 0 |  |

CDRT, candesartan; CVDL, carvedilol.

^*^P value calculated by Fisher’s exact test

**FIGURE S1** Subgroup analysis of candesartan in the change in LVEF during doxorubicin chemotherapy


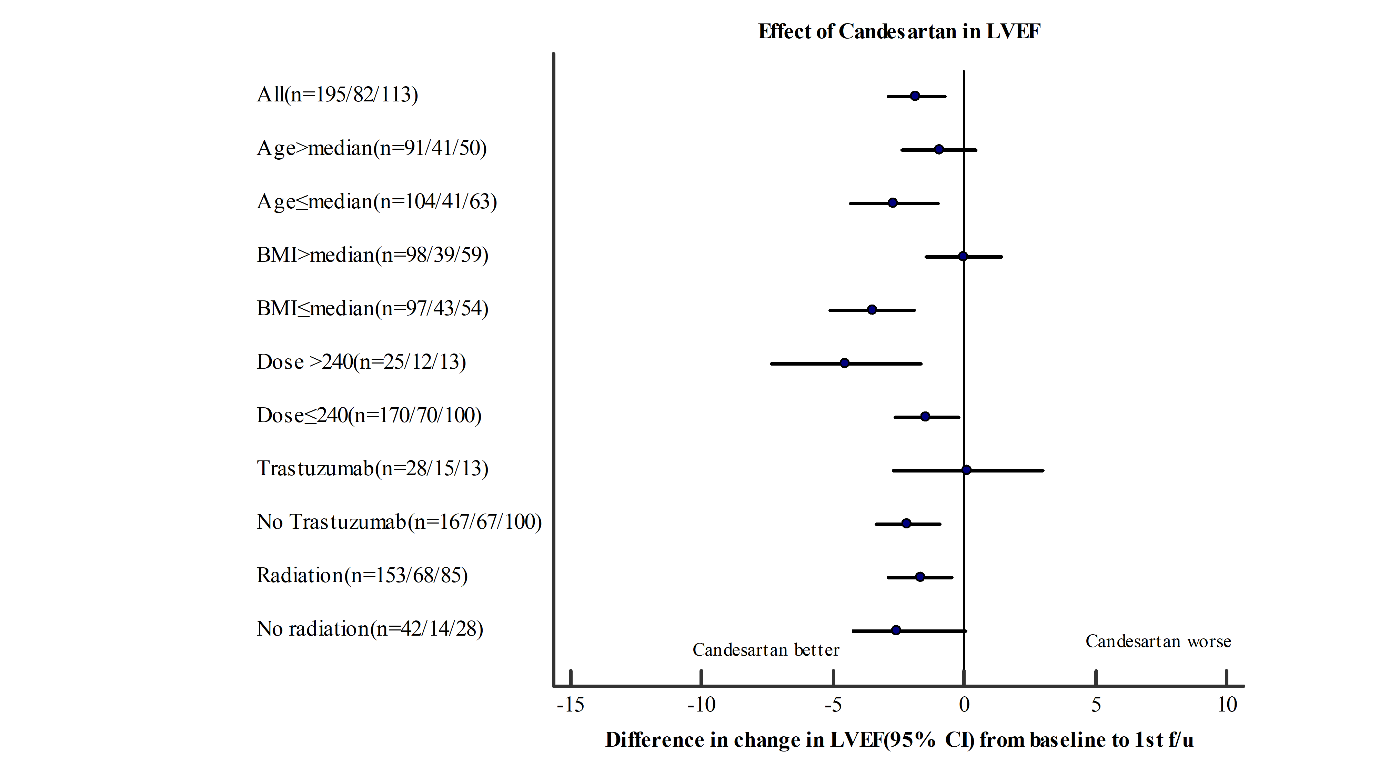


Effect of candesartan on changes in LVEF after chemotherapy expressed in percentage points with 95% confidence intervals. Concomitant treatment with candesartan reduced the decrease in LVEF, and this effect was observed across all subgroups.

LVEF, Left Ventricular Ejection Fraction; F/U, Follow-up; CI, Confidence Interval; BMI, Body Mass Index.

**FIGURE S2** Subgroup analysis of carvedilol in the change in LVEF during doxorubicin chemotherapy


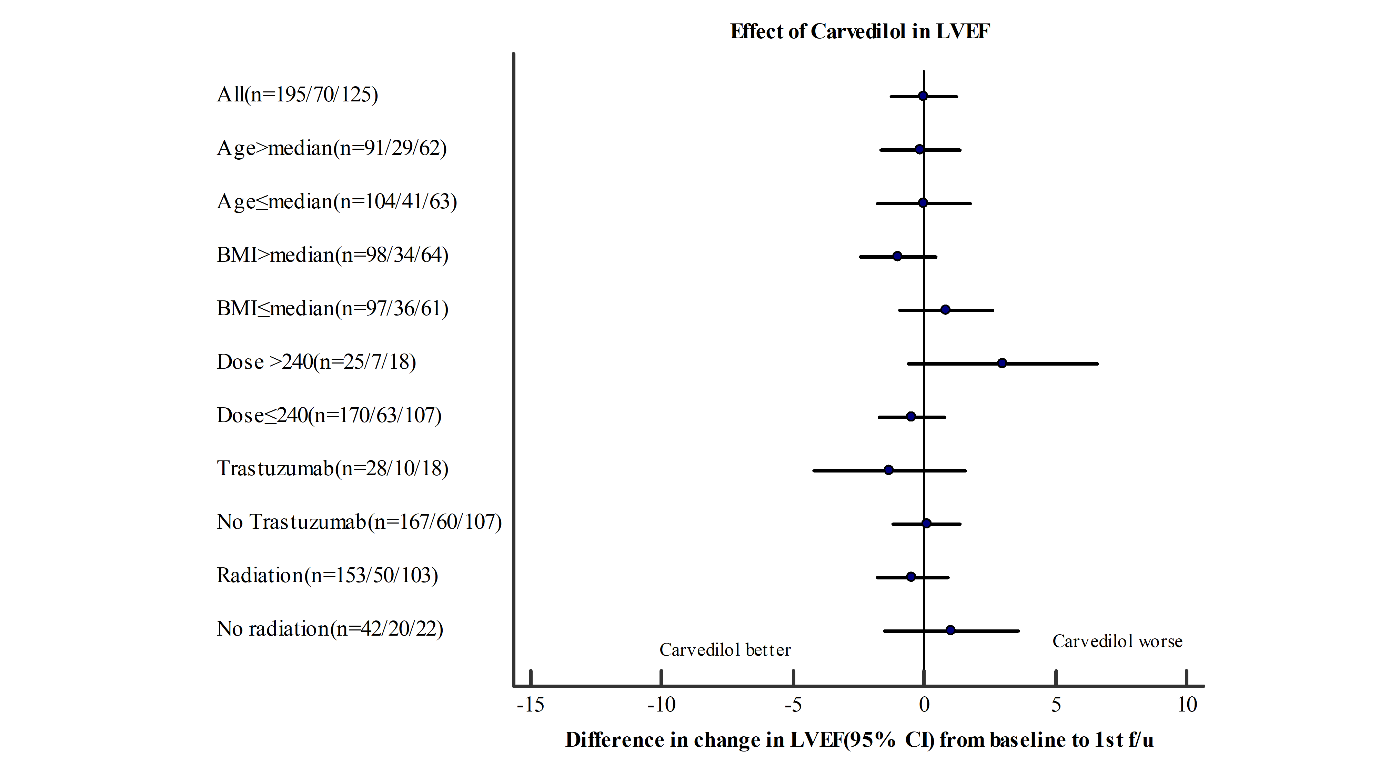


Effect of carvedilol on changes in LVEF after chemotherapy expressed in percentage points with 95% confidence intervals. Concomitant treatment with carvedilol showed inconsistent effects across subgroups.

LVEF, Left Ventricular Ejection Fraction; F/U, Follow up; CI, Confidence Interval; BMI, Body Mass Index.
